# Phylogeny and biogeography of the Japanese rhinoceros beetle, *Trypoxylus dichotomus* (Coleoptera: Scarabaeidae) based on SNP markers

**DOI:** 10.1002/ece3.6982

**Published:** 2020-12-22

**Authors:** Huan Yang, Chong Juan You, Clement K. M. Tsui, Luke R. Tembrock, Zhi Qiang Wu, De Po Yang

**Affiliations:** ^1^ School of Pharmaceutical Sciences Sun Yat‐Sen University Guangzhou China; ^2^ Beijing Key Laboratory for Forest Pest Control Beijing Forestry University Beijing China; ^3^ Department of Pathology Sidra Medicine Doha Qatar; ^4^ Department of Pathology and Laboratory Medicine Weill Cornell Medicine‐Qatar Ar‐Rayyan Qatar; ^5^ Division of Infectious Diseases Faculty of Medicine University of British Columbia Vancouver BC Canada; ^6^ Department of Agricultural Biology Colorado State University Fort Collins CO USA; ^7^ Shenzhen Branch Guangdong Laboratory for Lingnan Modern Agriculture Genome Analysis Laboratory of the Ministry of Agriculture Agricultural Genomics Institute at Shenzhen Chinese Academy of Agricultural Sciences Shenzhen China

**Keywords:** beetle taxonomy, genetic variation, island biogeography, phylogeography, population structure, SLAF‐seq

## Abstract

The Japanese rhinoceros beetle *Trypoxylus dichotomus* is one of the largest beetle species in the world and is commonly used in traditional Chinese medicine. Ten subspecies of *T. dichotomus* and a related *Trypoxylus* species (*T. kanamorii*) have been described throughout Asia, but their taxonomic delimitations remain problematic. To clarify issues such as taxonomy, and the degree of genetic differentiation of *Trypoxylus* populations, we investigated the genetic structure, genetic variability, and phylogeography of 53 specimens of *Trypoxylus* species from 44 locations in five Asian countries (China, Japan, Korea, Thailand, and Myanmar). Using specific‐locus amplified fragment sequencing (SLAF‐seq) techniques, we developed 330,799 SLAFs over 114.16M reads, in turn yielding 46,939 high‐resolution single nucleotide polymorphisms (SNPs) for genotyping. Phylogenetic analysis of SNPs indicated the presence of three distinct genetic groups, suggesting that the various subspecies could be treated as three groups of populations. PCA and ADMIXTURE analysis also identified three genetic clusters (North, South, West), which corresponded to their locations, suggesting that geographic factors were important in maintaining within population homogeneity and between population divergence. Analyses of SNP data confirmed the monophyly of certain subspecies on islands, while other subspecies (e.g., *T. d. septentrionalis*) were found to be polyphyletic and nested in more than one lineage. AMOVA demonstrated high level of differentiation among populations/groups. Also, pairwise *F*_ST_ values revealed high differentiation, particularly between South and West, as well as between North and South. Despite the differentiation, measurable gene flow was inferred between genetic clusters but at varying rates and directions. Our study demonstrated that SLAF‐seq derived markers outperformed 16S and COII sequences and provided improved resolution of the genetic differentiation of rhinoceros beetle populations from a large part of the species’ range.

## Graphical abstract

We estimate the evolution of morphological diversification as well as genetic differentiation, gene flow, and population genetic structure of Japanese rhinoceros beetle populations.

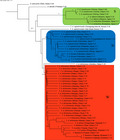

## 1 INTRODUCTION

The Japanese rhinoceros beetle, *Trypoxylus dichotomus* (Coleoptera, Scarabaeidae, Dynastinae) first described by Linnaeus (1771), is widely distributed in China, Japan, the Korean Peninsula, Vietnam, Myanmar, Laos, India, and Thailand (Adachi, 2017; Nagai, 2006, 2007; Satoru, 2014). It is typically found in broad‐leaved forests in tropical and subtropical mountainous habitats. The Japanese rhinoceros beetle has been important in Chinese traditional medicine for nearly 2000 years. Recent studies have found that extracts from the beetle have antihepatofibrotic, antineoplastic, and antibiotic effects (Chung et al., 2014; Kim et al., 2007; Miyanoshita et al., 1996; Ratcliffe et al., 2011; Sagisaka et al., 2001; Yoshikawa et al., 1999). A previously undescribed lectin purified from the larvae of Japanese rhinoceros beetles may assist in the suppression of human cervical cancer (HeLa), murine fibroblast (L929), and murine L1210 leukemic cells (Kui et al., 2000). Additional studies have found that Japanese rhinoceros beetles contain compounds with possible application as immunomodulators and tumor growth inhibitors (Hee et al., 2001; Ratcliffe et al., 2011; Umetsu et al., 1985, 1984). In addition to human medical applications, the bacterium *Bacillus amyloliquefaciens* KB3, isolated from feces of 3rd instar larvae of Japanese rhinoceros beetles, can be used as biocontrol against certain fungal plant pathogens (Nam et al., 2016). The beetle's high protein content gives it great potential as a food source when a suitable processing method is employed (Chung et al., 2013; Kim et al., 2016). In many East Asian countries, keeping Japanese rhinoceros beetles as pets has become a personal hobby and popular due to their large size and unique body shape.

Apart from the numerous applications, the Japanese rhinoceros beetle have received widespread attention owing mainly to their remarkable sexual dimorphism and individual variation, especially in the size and shape of the male's cephalic and pronotal horn (Buchalski et al., 2019; Hosoya & Araya, 2005; Ito et al., 2013; Morita et al., 2019; Ohde et al., 2018; Warren et al., 2013; Zinna et al., 2018). Male rhinoceros beetles have robust prominent cephalic horns for intraspecific competition over reproductive opportunities and in interspecific competition (like members of the *Lucanidae* family) over territory and resources. Horn shapes and sizes diverge greatly even among closely related species and sometimes between populations of beetles, suggesting differential selective pressures on horns among different lineages (Buchalski et al., 2019; Emlen et al., 2007, 2012; Hongo, 2003, 2007; Ito et al., 2013, Johns et al., 2014; Karino et al., 2005; Zinna et al., 2018). Studies on the genetic mechanism of horn development suggest that the genes underlying horn formation in rhinoceros beetles (Dynastinae) were the same genes recruited for the independent derivation of horns among the dung beetles (Scarabaeinae) (Ito et al., 2013; Ohde et al., 2018). As such rhinoceros beetles and related taxa are ideal for comparative studies of sexual selection, sexual dimorphism, and the evolution of morphological innovations.

**Figure 1:**
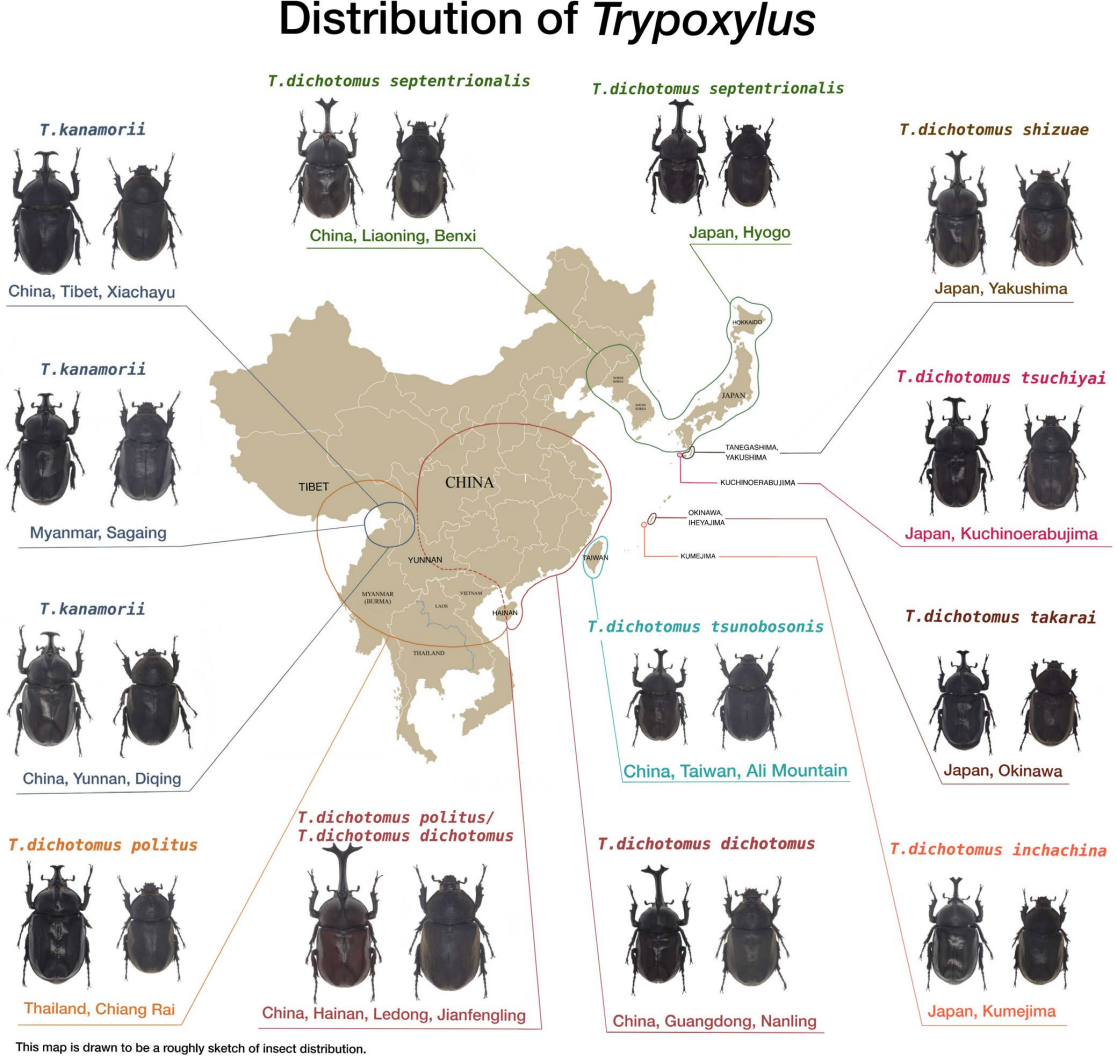
A map showing the distribution of *Trypoxylus* species in East Asia with illustrations of various subspecies

*T. dichotomus* was initially described by Linnaeus (1771) from specimens collected in Southeast Asia (at the time referred to as Indiis). In total, 10 subspecies of *T. dichotomus* along with another *Trypoxylus* species (*T. kanamorii* Nagai, 2006) were proposed in later revisions (Figure 1). The subspecies include *T. d. dichotomus* (Linnaeus, 1771) in Southeastern China; *T. d. inchachina* (Kusui, 1976) in Japan (Kumejima Island); *T. d. politus* (Prell, 1934) in Northeastern India, Myanmar, Thailand, Laos, and Vietnam; *T. d. septentrionalis* (Kôno, 1931) in Japan, the Korean peninsula, and Northeastern China; *T. d. tsunobosonis* (Kôno, 1931) in Taiwan; *T. d. takarai* (Kusui, 1976) in Japan (Okinawa Island and Iheyajima Island)*; T. d. tsunobosonis* Kôno in Taiwan; *T. d. tsuchiyai* (Nagai, 2006) in Japan (Kuchinoerabu‐jima Island); *T. d. shizuae* in Japan (Yakushima Island and Tanegashima Island); *T. d. xizangensis* (Li et al., 2015) in Tibet (China); *T. d. shennongjii* (Satoru, 2014) in China (Hubei Province) (Adachi, 2017; Kôno, 1931; Kusui, 1976; Li et al., 2015; Linnaeus, 1771; Nagai, 2006, 2007; Prell, 1934; Satoru, 2014); and the species *T.kanamorii* (Nagai, 2006) in Myanmar, India and China (Yunnan and Tibet). Species and subspecies division of *Trypoxylus* have been controversial, with traditional identifications mainly based on morphological characteristics, such as horn shape, body size and shape, lustrousness of elytra, and pubescence on the pygidium, pronotum, and elytra. Historically, delimitations among subspecies could be arduous because of limited divergence between the morphological characters used for subspecific identification. Later, Nagai (2006, 2007) proposed seven subspecies of *T. dichotomus* in Asia and illustrated differences among these subspecies based on morphological characteristics. Satoru (2014) and Adachi (2017) described additional *T. dichotomus* subspecies from Japan based on body color and pubescence on elytra. However, these studies have only employed a small number of diagnostic characters to separate the subspecies without placing the characters or taxa into a phylogenetic context to better understand character evolution within *Trypoxylus*. Taxonomy and distribution of *T. dichotomus* subspecies in Asia, especially in China, has also been challenging for many of the same reasons. As such, a molecular phylogenetic approach can provide much needed resolution in evaluating the monophyly of *T. dichotomus* subspecies that have been delimited with sometimes overlapping characters.

Understanding spatial distribution and species delineations is critical in clearly defining patterns of biodiversity and subsequently informing conservation decisions as well as providing lineage‐based comparisons for future studies, such as trait evolution and/or identifying novel medicinal compounds. The accurate inference of genetic clustering in natural beetle populations has important implications to understand the process and pattern of evolution. For instance, improved resolution of within‐species patterns of genetic diversity, divergence, and clustering can in turn improve estimates of population genetic processes, such as the resolution of spatial genetic differentiation among populations and increases accuracy and precision of gene flow rates estimates (Black & DuTeau, 1997; Zhu et al. 2018), which can be useful in identifying historic corridors of migration.

Numerous different molecular marker systems have been used to infer population structure and evolutionary patterns for a variety of insect species (e.g., Batista et al., 2016; Pascoal & Kilner, 2017). For instance, Pascoal and Kilner (2017) developed 14 polymorphic microsatellite markers and investigated levels of genetic differentiation in four populations of burying beetles (*Nicrophorus vespilloides*). With random amplified polymorphic DNA (RAPD) and mitochondrial DNA COII sequence analysis, homogeneity between small brown planthoppers from different geographic regions could be inferred (Xu et al., 2001; Wang et al. 2019). Single nucleotide polymorphisms (SNPs) from genome‐wide panels are known to improve the resolution of population genetic inferences such as linkage disequilibrium (LD) and population structure because of the abundant and generally even distribution across the genome over other markers, and the well‐understood models of evolution applied to nucleotide mutation analyses. The use of SNPs in studies dissecting complex traits is also attractive because they can be used to quantify heterozygosity in biallelic loci, they can be sequence‐tagged to ensure orthologous comparison of loci, and genotyping can be massively multiplexed to increase throughput and reduce cost (Blanco‐Bercial & Bucklin, 2016). High‐throughput next‐generation sequencing (NGS) technologies are now critical tools in developing SNP panels and genotyping subsequent individuals in studies of the ecology and evolution of traditionally poorly resolved cryptic groups. An example of a successful NGS method for large‐scale de novo SNP discovery and genotyping is SLAF‐seq (specific length amplified fragment sequencing) developed by Sun et al. (2013). The SLAF‐seq method has proven efficient and effective for SNP based population genetic analyses in numerous plant and animal studies (Su et al., 2017; Sun et al., 2013; Wang et al. 2019).

To better understand the evolution of morphological diversification as well as clarify genetic differentiation, gene flow, and population genetic structure of Japanese rhinoceros beetle populations, we conducted phylogenetic and population genetic analyses across 10 subspecies from a large portion of the species range in eastern Asia. DNA sequences from the mitochondrial cytochrome c oxidase subunit II (COII) and 16S ribosomal RNA (rRNA) genes were employed in reconstructing the phylogeny of *Trypoxylus* species and subspecies. SLAF‐seq derived SNPs were also used in population genetic and phylogenetic analyses for comparison to sequence‐based datasets and for improved resolution of inference.

## 2 MATERIALS AND METHODS

### 2.1 Insect specimen collection

During the season of peak activity (from May to mid‐August) in the years 2017 to 2018, live *Trypoxylus* individuals were caught by light trap or collected on sap exudate (food source for beetles) of broad‐leaved trees. A total of 128 individual beetles from 44 locations in five different regions of Asia (19 provinces in China, seven locations in Japan, two locations in Korea, one location in Thailand, and one in Myanmar) were obtained in this study as well as outgroup taxa included two beetles of *Xylotrupes mniszechi* from Tibet (China) and *Xyloscaptes davidis* from Vietnam. 53 specimens of *Trypoxylus* belonging to two species (*T. kanamorii* and *T. dichotomus*) and nine subspecies from five regions of Asia were selected for SLAF‐seq analysis and phylogenetic analysis based on 16S and COII sequences (Table 1). Specimens from the two subspecies, *T. d. shennongjii* and *T. d. xizangensis,* could not be obtained. Immediately upon collection, beetles were preserved in absolute ethyl alcohol. Thereafter, collected specimens were mounted, morphological observations recorded, and digitally imaged by Nikon D7000 camera. Previous to these steps, the thoracic muscles were dissected for examination, preserved in absolute ethyl alcohol, and stored at −20°C until later use in DNA extractions. All examined voucher specimens were deposited in the School of Pharmaceutical Sciences, Sun Yat‐sen University (Guangzhou, China).

**Table 1:** Sampling information of *Trypoxylus* specimens used in this investigation

Cluster	Species	Location	Code	GenBank accession no. 16S	GenBank accession no. COII
North	*T. d. septentrionalis*	Tonghua, Jilin, China (JL)	T‐75	MT080160	MT103637
	*T. d. septentrionalis*	Dandong, Liaoning, China (LN1)	T‐50	MT080148	MT103651
	*T. d. septentrionalis*	Benxi, Liaoning, China (LN2)	T‐52	MT080164	MT103632
	*T. d. septentrionalis*	Gyungsangnum‐do, Korea (KO1)	T‐22	MT080121	MT103623
	*T. d. septentrionalis*	Fukushima, Japan (FUK1)	T‐7	MT080129	MT103638
	*T..d septentrionalis*	Fukushima, Japan (FUK2)	T‐8	MT080132	MT103631
	*T. d. septentrionalis*	Kuriyama, Hokkaido, Japan (KUR1)	T‐6	MT080141	MT103640
	*T. d. septentrionalis*	Kuriyama, Hokkaido, Japan (KUR2)	T‐5	MT080157	MT103643
	*T. d. septentrionalis*	Hyogo, Japan (HYO)	T‐1	MT080137	MT103621
	*T. d. shizuae*	Yakushima, Japan (YAK1)	T‐11	MT080155	—
	*T. d. shizuae*	Yakushima, Japan (YAK2)	T‐12	MT080136	MT103633
	*T. d. tsuchiyai*	Kuchinoerabu‐jima, Japan (KUC1)	T‐13	MT080133	MT103608
	*T. d. tsuchiyai*	Kuchinoerabu‐jima, Japan (KUC2)	T‐14	MT080134	MT103636
	*T. d. septentrionalis*	Jeju Island, Korea (JEJ)	T‐25	MT080124	MT103613
	*T. d. dichotomus*	Guiyang, Guizhou, China (GUI)	T‐38	MT080119	MT103634
West	*T. d. dichotomus*	Fuzhou, Jiangxi, China (JX1)	T‐31	MT080131	MT103647
	*T. d. dichotomus*	Xinyu, Jiangxi, China (JX2)	T‐34	MT080135	MT103652
	*T. d. dichotomus*	Lishui, Zhejiang, China (ZJ1)	T‐76	MT080126	MT103641
	*T. d. dichotomus*	Wuyishan, Fujian, China (FJ1)	T‐27	MT080161	MT103642
	*T. d. dichotomus*	Nanling, Guangdong, China (GD)	T‐65	MT080127	MT103624
	*T. d. dichotomus*	Zhumadian, Henan, China (HEN1)	T‐41	MT080156	MT103622
	*T. d. dichotomus*	Xiangyang, Hubei, China (HB)	T‐49	MT080114	MT103630
	*T. d. dichotomus*	Zhangzhou, Fujian, China (FJ2)	T‐28	MT080159	MT103639
	*T. d. dichotomus*	Nanchang, Jiangxi, China(JX3)	T‐32	MT080149	MT103635
	*T. d. dichotomus*	Meishan, Sichuan, China (SC1)	T‐57	MT080163	MT103609
	*T. d. dichotomus*	Deyang, Sichuan, China (SC2)	T‐59	MT080113	MT103653
	*T. d. dichotomus*	Ningshan, Shaanxi, China (SX)	T‐54	MT080139	MT103626
	*T. d. dichotomus*	Chizhou, Anhui, China (AH)	T‐73	MT080138	MT103616
	*T. d. dichotomus*	Suqian, Jiangsu, China (JS1)	T‐47	MT080147	MT103604
	*T. d. dichotomus*	Yangzhou, Jiangsu, China (JS2)	T‐45	MT080158	MT103625
	*T. d. dichotomus*	Huzhou, Zhejiang, China (ZJ2)	T‐78	MT080125	MT103629
	*T. d. dichotomus*	Nanyang, Henan, China (HEN2)	T‐39	MT080120	MT103607
	*T. d. dichotomus*	Jiujiang, Jiangxi, China (JX4)	T‐36	MT080115	MT103610
	*T. d. dichotomus*	Suzhou, Jiangsu, China (JS2)	T‐43	MT080146	MT103649
	*T. d. dichotomus*	Changde, Hunan, China (HN)	T‐70	MT080128	MT103612
	*T. d. dichotomus*	Dayaoshan, Guangxi, China (GX)	T‐60	MT080142	MT103648
	*T. d. dichotomus*	Wuzhishan, Hainan, China (HAN1)	T‐67	MT080112	MT103605
	*T. d. dichotomus*	Jianfengling, Hainan, China（HAN2)	T‐69	MT080130	MT103614
	*T. kanamorii*	Xiachayu, Tibet, China (TB)	T‐79	MT080140	MT103655
	*T. d. politus*	Xishuangbanna, Yunnan, China (YN1)	T‐64	MT080143	MT103644
	*T. kanamorii*	Diqing, Yunnan, China (YN2)	T‐62	MT080144	MT103645
	*T. d. politus*	Wiang Papau, Thailand (THA1)	T‐17	MT080118	MT103618
	*T. d. politus*	Wiang Papau, Thailand (THA2)	T‐18	—	—
	*T. kanamorii*	Sagaing, Myanmar (MYA1)	T‐15	MT080117	MT103615
	*T. kanamorii*	Sagaing, Myanmar (MYA2)	T‐16	MT080145	MT103646
South	*T. d. septentrionalis*	Gyungsang num‐do, Korea (KOR2)	T‐20	MT080122	MT103650
	*T. d. tsunobosonis*	Pingtung, Taiwan (TW1）	T‐81	MT080150	MT103627
	*T. d. tsunobosonis*	Tengjhih, Taiwan (TW2）	T‐80	MT080116	MT103620
	*T. d. tsunobosonis*	Taichung, Taiwan (TW3）	T‐82	MT080151	MT103619
	*T. d. takarai*	Okinawa, Japan (OKI1)	T‐10	MT080154	MT103606
	*T. d. takarai*	Okinawa, Japan (OKI2)	T‐9	MT080110	MT103602
	*T. d. inchachina*	Kumejima Island, Japan (KUM1)	T‐3	MT080152	—
	*T. d. inchachina*	Kumejima Island, Japan (KUM2)	T‐4	MT080153	MT103611
Outgroup	*Xylotrupes mniszechi*	Chayu, Tibet, China (TB2)	T‐84	MT080111	MT103603
	*Xyloscaptes davidis*	Vietnam (VIET1)	T‐85	MT080162	MT103654

### 2.2 Morphological data

All specimens in our study were identified based on morphological characters published in previous studies (Adachi, 2017; Kôno, 1931; Kusui, 1976; Li et al., 2015; Nagai, 2006, 2007; Prell, 1934; Satoru, 2014). Some morphological characters, including body size, lustrousness, cuticle color, cephalic and pronotal horn size, pubescence on elytra, pronotum, head, and pygidium, have provided useful criteria for species identification (Table 2). In this study, the taxonomic revisions by Nagai (2006, 2007), Satoru (2014), and Adachi (2017) were followed as they provided the clearest subspecies descriptions. The genus *Xylotrupes* was treated as an outgroup in our comparisons and thought be a more distantly related to *Trypoxylus* than *Xyloscaptes* (also used as an outgroup) based on Dutrillaux et al. (2013). Digital images of diagnostic features were taken with a Nikon D7000 camera with AF‐S VR Micro‐Nikkor 105mm f/2.8G IF‐ED‐Nikon lenses and edited with Adobe Photoshop CS4, Microsoft Paint, and Autodesk Sketchbook.

**Table 2:** Morphological features of *Trypoxylus* species in this investigation

Species	Locations	Descriptions	Resource	Genetic group
*T. kanamorii*	Northwest of Myanmar; India, Tibet (China);Yunnan (China)	♂: slender and dark brown to black body; strongly lustrous elytra; shorter horns of head and pronotum ♀: very slight pubescence on elytra compared to *T. d. septentrionalis* in Japan	Nagai (2006, 2007)	West
*T. d. dichotomus*	Middle and South of China	Bigger body size and slender body shape; weak lustrous elytra compared to *T. d. septentrionalis* in Japan; lack of detailed description when first described as *Scarabaeus* by Linnaeus	Linnaeus (1771), Nagai (2007)	West
*T. d. inchachina*	Kumejima Island (Japan), Ryukyu Islands (Japan)	♂: entirely black; very short forked cephalic horn with each apex weakly bifurcated and very short abruptly bifurcated pronotal horn; shorter legs; smaller body, strongly lustrous elytra; smaller cephalic horns compared to *T. d. septentrionalis* in Japan; slightly less lustrous than *T. d. politus* from Thailand; ♀: prothorax apical margin angulately sinuate at middle; head middle tubercle slightly lower than other outer ones; longitudinal hollow of prothorax shallow and not bifurcated near apical margin	Kusui (1976), Nagai (2007)	South
*T. d. politus*	Myanmar, Thailand, Vietnam, Yunnan (China) and Laos	First described in Laos as having glossier body due to sparse pubescence and dark body color; shorter, wider body; ♂: strongly lustrous elytra compared to *T. d. septentrionalis* in Japan; ♂: most lustrous body among all subspecies	Nagai (2007), Prell (1934)	West
*T. d. septentrionalis*	Northeast of China; Korean Peninsula; Jeju Island; Japan (Honshu, Shikoku, Kyushu, Hokkaido, and some other small islands of Japan)	Robust body; ♂: weak lustrous elytra; sparse pubescence on pygidium	Kôno (1931), Nagai (2007)	North
*T. d. shennongjii*	Shennongjia, Hubei (China)	♂: a large and magnificent V‐shaped cephalic horn, which has a small fork on the apex	Satoru (2014)	—
*T. d. shizuae*	Yakushima Island and Tanegashima Island (Japan)	Brighter reddish‐brown body, sometimes dark brown; robust body; slender front tibiae; ♂: shorter, convex pronotum; shorter cephalic and pronotal horn compared to *T. d. septentrionalis*; rounded pronotal lateral margin and curved gradually at the former point; fewer, shorter, and finer pubescence on metasternum, second abdominal sternite, and abdomen, ♀: faintly lustrous; otherwise similar to *T. d. septentrionalis* in Japan	Adachi (2017)	North
*T. d. takarai*	Mainland of Okinawa; Ryukyu Islands (Japan)	Smaller body size; ♂: smaller cephalic horn; darker body color; shorter pubescence on center of elytra and pronotum, therefore strongly lustrous compared to *T. d. septentrionalis* in Japan; ♀: middle tubercle of head lower or similar to outer ones in height; longitudinal hollow of prothorax not distinctly bifurcate near apical margin	Kusui (1976), Nagai (2007)	South
*T. d. tsuchiyai*	Kuchinoerabu‐jima Island (Japan)	Smaller and wider body than *T. d. septentrionalis*; naked or clothed with pubescence on pronotum; smoother and shining elytra; shorter and coarser pubescence on pygidium otherwise similar to *T. d. septentrionalis* in Japan	Nagai (2006, 2007)	North
*T. d. tsunobosonis*	Taiwan	♂: thinner but longer pronotal horn compared to *T. d. septentrionalis* in Japan.	Kôno (1931), Nagai (2007)	South
*T. d. xizangensis*	Gamdo City, Tibet (China)	♂: Thin horn; sharp‐pointed lateral horn; short and thick phallobase	Li et al. (2015)	—

### 2.3 DNA extraction, PCR, and sequencing of 16S and COII

Genomic DNA was extracted from thoracic muscle tissue using the QIAGEN DNeasy Tissue Kit in accordance with manufacturer's instructions. For sequence‐based phylogenetic analysis, amplicons from cytochrome c oxidase II (COII) and 16S ribosomal RNA (16S rRNA) were used as they are common loci in barcoding. The 16S rRNA region was amplified by two primer sets (16sF/16sR and 16SB/16SA) (16sF: 5′‐CGCCTGTTTATCAAAAACAT‐3′; 16sR: 5′‐CTCCGGTTTGAACTCAGATCA‐3′), (16SA: 5′‐CGCCTGTTTAACAAAAACATGT‐3′; 16SB: 5′‐CCGGTTTGAACTCAGATCATGT‐3′), which were employed because they successfully amplified species in *Lucanidae* (Han et al., 2010; Rowland & Miller, 2012). The COII region was amplified by primers F‐lue and R‐lys (F‐lue: 5′‐TCTAATATGGCAGATTAGTGC‐3′; R‐lys: 5′‐GAGACCAGTACTTGCTTTCAGTCATC‐3′; Rowland & Miller, 2012). PCR reactions were performed in 25 μl volumes containing 1 μl DNA template, 2.5 μl forward primer (2 μM), 2.5 μl reverse primer (2 μM), 12.5 μl of 2× EsTaq Master Mix (Cwbio), and 6.5 μl ddH_2_O. The PCR thermocycle conditions were as follows: 94°C for 3 min; followed by 35 cycles at 95°C for 1 min, 40–50°C (depending on primer set) for 1 min, and 72°C for 1 min; and a final extension step of 72°C for 10 min. The PCR products were purified and visualized by gel electrophoresis on 1.5% agarose gel to confirm amplification. Sanger sequencing was performed at TSINGKE. All sequences are available from GenBank under accession numbers MT103602–MT103655 for COII and MT080110–MT080164 for 16S rRNA.

### 2.4 Phylogenetic analysis

16S and COII sequences were edited using Seqman v. 7.1.0 in the DNASTAR Lasergene core suite software (DNASTAR Inc., Madison, WI, USA), and aligned using MAFFT v.6 (Katoh & Standley, 2013). Ambiguously aligned sequences were excluded from the analysis.

Maximum parsimony (MP) analysis of concatenated 16S and COII alignment was performed in PAUP v.4.0b10 (Swofford, 2003). Gaps were treated as missing data, and all characters were equally weighted. Trees were inferred using the heuristic search option with TBR branch swapping. The robustness of the most parsimonious trees was evaluated by 1,000 bootstrap replications (Felsenstein, 1985). Other calculated measures were the tree length (TL), consistency index (CI), retention index (RI), and rescaled consistency (RC).

Bayesian analyses (BI) were performed using MrBayes v.3.1.2 with Markov chain Monte Carlo (MCMC) and Bayesian posterior probabilities (Ronquist & Huelsenbeck, 2003). Default parameters were selected, and the evolutionary model was set to the GTR + I + G model, which was the best model predicted for the concatenated 16S and COII alignment by MrModeltest v. 2.3 (Nylander, 2004). Simultaneous Markov chains were computed for 1,000,000 generations, and the trees were sampled every 100th generation (You et al., 2019).

Maximum likelihood analysis (ML) was performed by RAxMl v. 1.3 (Stamatakis, 2006). The bootstrapping was carried out with 1,000 replicates using the GTR + I + G model. *Xylotrupes mniszechi* and *Xyloscaptes davidis* were selected as outgroup taxa.

### 2.5 SLAF library construction and high‐throughput sequencing

For SLAF‐seq analysis, DNA concentration was quantified using a NanoDrop‐2000 spectrophotometer, and all DNA samples were diluted to 50 ng/μl. SLAF library construction was carried out following Sun et al. (2013) with minor modifications. To obtain evenly distributed SLAF tags and to avoid repetitive SLAF tags for maximum SLAF‐seq efficiency, simulated restriction enzyme digestion was carried out in silico. Genomic DNA was digested using *RsaI‐HaeIII* restriction enzyme, and the reference genome of *Dendroctonus ponderosae* (https://www.ncbi.nlm.nih.gov/genome/?term=Dendroctonus+ponderosae) was used to predict enzyme digestion. DNA fragments of 264–364 bp were selected as SLAFs and prepared for paired‐end sequencing on the Illumina High‐Seq 2,500 sequencing platform (Illumina, Inc.) at Biomarker Technologies Corporation.

### 2.6 SLAF‐seq data grouping and genotyping

Raw pair‐end reads were clustered based on sequence similarity. Sequences with over 90% identity were grouped in one SLAF tag, SLAFs with low‐depth coverage were filtered out (Huang et al., 2016; Sun et al., 2013; Wang et al. 2019). Only groups with higher depth and four tags or fewer were identified as high‐quality SLAFs with SLAFs possessing two, three, or four tags identified as polymorphic. In this study, depth was 17.63× on average, and a total of 1,374,985 high‐quality unique SLAF tags were obtained with 330,799 of those tags considered polymorphic.

Development of SNP markers was based on reference sequence with very high depth in each SLAF tag. SAMtools and GATK were used for mapping and SNP calling (Li et al., 2009; McKenna et al., 2010; Wang et al. 2019). A total of 46,939 SNPs with minor allele frequencies (MAF) of ≥0.05 and an integrity score of ≥80% were employed in downstream analyses.

### 2.7 SNP Data analysis

#### 2.7.1 Population genetics analyses

Phylogenetic trees based on a polymorphic SNP matrix (53 individuals × 46,939 SNPs, also used in all subsequent analyses unless noted otherwise) were constructed using the neighbor joining (NJ) method employed in MEGA X with p‐distance and pairwise deletion option parameters (Kumar et al., 2018; Saitou & Nei, 1987; Tamura et al., 2011). Bootstrap tests with 1,000 replicates were applied to assess branch support in the final consensus trees. Bayesian Inference (BI) was performed using MrBayes 3.1.2 (Ronquist & Huelsenbeck, 2003) with Markov chain Monte Carlo (MCMC) and Bayesian posterior probabilities to assess branch support. Default parameters were selected, using a GTR (general time reversible) substitution model with gamma‐distributed rate variation across sites and a proportion of invariable sites (Ronquist & Huelsenbeck, 2003). Simultaneous Markov chains were run for 1,000,000 generations, and with sampling every 100th generation (You et al., 2019).

#### 2.7.2 Population structure

Population structure and genetic clustering of the 53 specimens was inferred using ADMIXTURE (Alexander et al., 2009), which does so through running population expansion models as well as inferring evolutionary and ecological processes among populations. ADMIXTURE is a model‐based clustering method used to analyze the association of individuals from multilocus SNP genotypes and infer clusters to test for population admixture. A range of cluster values (K) from 1 to 10 was assessed, with each run set to allow admixture and with a correlated allele frequency model, using the first 100,000 MCMC generations as burn‐in followed by 900,000 MCMC generations. The clustering results were cross‐verified, and the optimal number of clusters (K) was determined according to the lowest value of the cross‐validation error rate between cluster values (Debnath, 2014; Wang et al. 2019). A nonmodel‐based principal components analysis (PCA) was performed with EIGENSOFT v. 6.0, to further assess clustering of genotypes (Price et al., 2006).

### 2.8 Genetic diversity

#### 2.8.1 Polymorphism analysis

Power Marker (Liu and Muse, 2005) was used to calculate measures of genetic diversity: observed and expected allele number, observed and expected heterozygosity, Nei's diversity index, Shannon–Weiner index, and polymorphism information content. Observed (*H*_o_) and expected heterozygosity (*H*_e_) were defined as the probability that two randomly chosen alleles from the population are different (Nei, 1978). The polymorphism information content (PIC) value is commonly used as an estimate of the probability of finding polymorphism between two random samples (Shete et al., 2000).

#### 2.8.2 Genetic differentiation and gene flow between genetic clusters

Genetic differentiation was calculated using an analysis of molecular variance (AMOVA) as applied in ARLEQUIN v. 3.5.1.2, which is a method of quantifying at what level (individual, population, or total) genetic variation is greatest (Excoffier & Lischer, 2010; Peakall & Smouse, 2005; Tsui et al., 2014, 2012). The divergence index and F‐statistics (*F*_ST_) were used to measure the level of genetic differentiation among *T. dichotomus* populations based on genetic polymorphism in the SNP data (Hudson et al., 1992), and *F*_ST_ was calculated via the PopGen 32 package in BIOPERL (Yeh et al., 1997) using a 100‐kb sliding windows in 10‐kb steps. Pairwise *F*_ST_ values were evaluated using a randomization test with 1,000 iterations using ARLEQUIN v. 3.5.1.2 (Excoffier & Lischer, 2010; Tsui et al., 2014, 2012).

The direction and rate of migration between genetic clusters (derived from the results of ADMIXTURE) were inferred using MIGRATE‐N v. 3.6.4 (Beerli & Palczewski, 2010), which uses an expansion of the coalescent theory to estimate migration rates. Four possible models for migration between each genetic cluster were evaluated (Yang et al., 2018). The number of recorded steps in the chain was set to 500,000, and the models were run three separate times to confirm convergence of parameter estimates. The marginal likelihoods of all models were compared to infer the direction and rate of gene flow, and only the results of the run that yielded the highest Bezier approximation score (1b) value were presented (Han et al., 2016; Tsui et al., 2014, 2012; Yang et al., 2018).

## 3 RESULTS

### 3.1 Morphology

External morphology of all *Trypoxylus* subspecies were observed and recorded (Table 3). Morphological diagnostic features for delimiting taxa were compared by taxa and geographic origin to assess the degree of overlap or separation of each character. Based on morphological criteria, these *Trypoxylus* samples included four *T. kanamorii* from Yunnan (China), Tibet (China), Sagaing (Myanmar); 24 *T. d. dichotomus* from Southern China; 11 *T. d. septentrionalis* from Northeastern China, Korean Peninsula, Jeju Island (Korea), Hokkaido (Japan), Hyogo (Japan), and Fukushima (Japan); three *T. d. politus* from Yunnan (China) and Wiang Papau (Thailand); two *T. d. shizuae* from Yakushima (Japan); three *T. d. tsunobosonis* from Taiwan; two *T. d. takarai* from Okinawa (Japan); and two *T. d. tsuchiyai* from Kuchinoerabu‐jima (Japan).

**Table 3:** Additional morphological descriptions of *Trypoxylus* specimens in different regions

Specimens	Features	♂	♀
Hainan Island (China)	Cuticle coloration is dark wine red in most specimens. Wider, robust body. Morphology and distribution are similar to *T. d. politus* in Vietnam		Elytra and pronotum highly lustrous in males due to sparse pubescence. Long robust cephalic horn in large males. large males commonly come to light trap in tropical rain forests but rarely found on trees by collectors
Taiwan	Commonly identified to be *T. d. tsunobosonis* where *tsunobosonis* implies their thin pronotal horn in Latin. Cuticle coloration ranges from dark red to dark orange. Thinner pronotal horn compared to *T. d. septentrionalis* in Japan. Slightly smaller on average than *T. d. dichotomus* from mainland China. Large body size individuals are common, commonly and easily found on broad‐leaved trees across Taiwan thereby light trap is not needed		
Mainland Okinawa (Japan)	Commonly identified to be *T. d. takarai*. Dark red cuticle coloration, smaller body size, smaller cephalic and pronotal horn size, shorter pubescence on center of elytra and pronotum therefore strongly lustrous compared to *T. d. septentrionalis* in Japan. similar to *T. d. inchachina* from Kumejima (Japan). Slightly thinner pronotal horn than *T. d. inchachina* in most individuals		
Kumejima (Japan)	Commonly identified to be *T. d. inchachina*. Dark red cuticle coloration, smaller body size, strongly lustrous elytra, smaller cephalic horns compared to *T. d. septentrionalis* in Japan. Slightly more lustrous than *T. d. takarai* and less lustrous than *T. d. politus* from Thailand. Slightly shorter and thicker pronotal horn than *T. d. takarai* in most individuals. Unlike *T. d. septentrionalis* from Japan, population on Kumejima are collected in deep jungles that are guarded by military force which is out of most local collectors’ collecting range		
Kuchinoerabu‐jima (Japan)	Commonly identified to be *T. d. tsuchiyai*. Smaller and wider body, naked or clothed with pubescence on pronotum, shorter and coarser pubescence on pygidium compared to *T. d. septentrionalis* in Japan. Smaller average body size, smoother and shining lustrous elytra otherwise similar to *T. d. septentrionalis* in Japan. *T. d. shizuae*, *T. d. tsuchiyai* both lined similar to *T. d. septentrionalis* in morphology, genetics, and geology, these two subspecies were established mostly evidential to their island distribution and slight differences in their morphological characteristics		
Yakushima (Japan)	Commonly identified to be *T. d. shizuae*. Brighter reddish‐brown, sometimes dark brown cuticle coloration, robust body, slender front tibiae, elytra strongly convex, shorter pronotum, rounded pronotal lateral margin and curved gradually at the former point	Shorter cephalic horn compared to *T. d. septentrionalis*	Faintly lustrous, fewer, shorter and finer pubescence on metasternum, second abdominal sternite, and abdomen compared to *T. d. septentrionalis* in Japan but very similar. *T. d. shizuae*, *T. d. tsuchiyai* both lined similar to *T. d. septentrionalis* in morphology, genetics, and geology, these two subspecies were established mostly evidential to their island distribution and slight differences in their morphological characteristics
Honshu (Japan), Shikoku (Japan), Kyushu (Japan), and Hokkaido (Japan)	Commonly identified to be *T. d. septentrionalis*. Cuticle coloration ranges from bright red to dark red, large body size is commonly observed, commonly captured on broad‐leaved trees and by light trap, robust body, weak lustrous elytra, sparse pubescence on pygidium. Population in Hokkaido was introduced by human in recent years due to pet importation, and the population is still growing in Hokkaido every year, imagines collected by light trap or on host trees, sometimes collecting larvae by chopping logs at timber piles near forests		
Korea, Liaoning (China), Jilin (China)	Commonly identified to be *T. d. septentrionalis*. Cuticle coloration ranges from dark red to black, darker than *T. d. septentrionalis* in Japan. Large body size is commonly observed, commonly captured on broad‐leaved trees and by light trap, sometimes collecting larvae by chopping logs at timber piles near forests, robust body, weak lustrous elytra, sparse pubescence on pygidium		
Zhangzhou, Fujian (China)	Commonly identified to be *T. d. dichotomus*. All three male specimens examined have similar small size, thinner pronotal horn, and dark body color. Genitals and geology are also close to *T. d. tsunobosonis* in Taiwan, yet genetic data clusters it together with the rest of *T. d. dichotomus* in China		
Mt. Tianmushan, Linan, Zhejiang (China)	Commonly identified to be *T. d. dichotomus*. It should be noted that specimens caught on Mt. Tianmushan are significantly smaller in average body size compared with other specimens across China		
Southeast of mainland China	Commonly identified to be *T. d. dichotomus*. Cuticle coloration ranging from dark red to bright red, commonly bigger body size, slender body shape, weak lustrous elytra compared to *T. d. septentrionalis* in Japan		
Sichuan (China) and Shaanxi (China)	Commonly identified to be *T. d. dichotomus*. Large body size	Cuticle coloration is darker, ranging from black to dark red, robust cephalic horn	
Guangxi (China)	Cuticle coloration is bright wine red.		Highly lustrous elytra and pronotum in males, similar to *T. d. politus* from Vietnam in morphology and geology. Lustrousness greater than specimens collected in Hainan but color is more reddish
Tibet (China), Diqing Yunnan (China), and Sagaing (Myanmar)	Commonly identified to be *T. kanamorii*. Smaller body size, cuticle color black, slender body	Strongly lustrous elytra, shorter horns of head and pronotum, very slight pubescence on elytra in female, commonly found in higher elevation than other *T. dichotomus* subspecies. Its morphological difference is likely to be an adaptation to its higher elevation distribution habitats, male individuals are very similar to *T. d. politus* in Thailand	
Xishuangbanna (Yunnan) and Thailand	Commonly identified to be *T. d. politus* where *politus* describes their lustrous elytra and pronotum in Latin, medium to large body size, dark cuticle color ranging from dark red to commonly seen black	Most lustrous elytra and pronotum among all subspecies, shorter and wider body, commonly imported to China, Taiwan, Japan, and Korea as pets due to the fact that *T. d. politus* imagines do not have stinky smells like *T. d. dichotomus* from China and *T. d. septentrionalis* from Japan, usually collected by light trap	
Vietnam and Laos	Commonly identified to be *T. d. politus*. Large body size, cuticle color ranging from dark red to black	Lustrous elytra and pronotum	
Dibang (India)	Commonly identified close to *T. kanamorii* or *T. d. politus*. Lighter culicle color (dark reddish‐brown to black) than *T. kanamorri* from Myanmar, small pronotal horn and cephalic horn, small body size; rounded body shape	Lustrous elytra and pronotum, sparse pubescence on pronotum and elytra in female, commonly collected by light trap at altitude above 2,000 m, distribution close to Chayu, Tibet, China	

The coloration of cuticle ranges from black to red; body length, including horn, ranges from 3 cm to over 9 cm, and all other external morphological characters had similarly broad and sometimes overlapping values across regions. Similarly, none of the analyzed morphological characters (commonly used in the *Trypoxylus* taxonomy) was individually proved to be unambiguously species‐ or subspecies‐specific. As such, additional morphological characters should be used in the further taxonomic study of *Trypoxylus*. For example, male genitalia have been successfully employed to delimit numerous Coleopteran species.

### 3.2 Phylogenetic analysis based on 16S and COII

**Figure 2:**
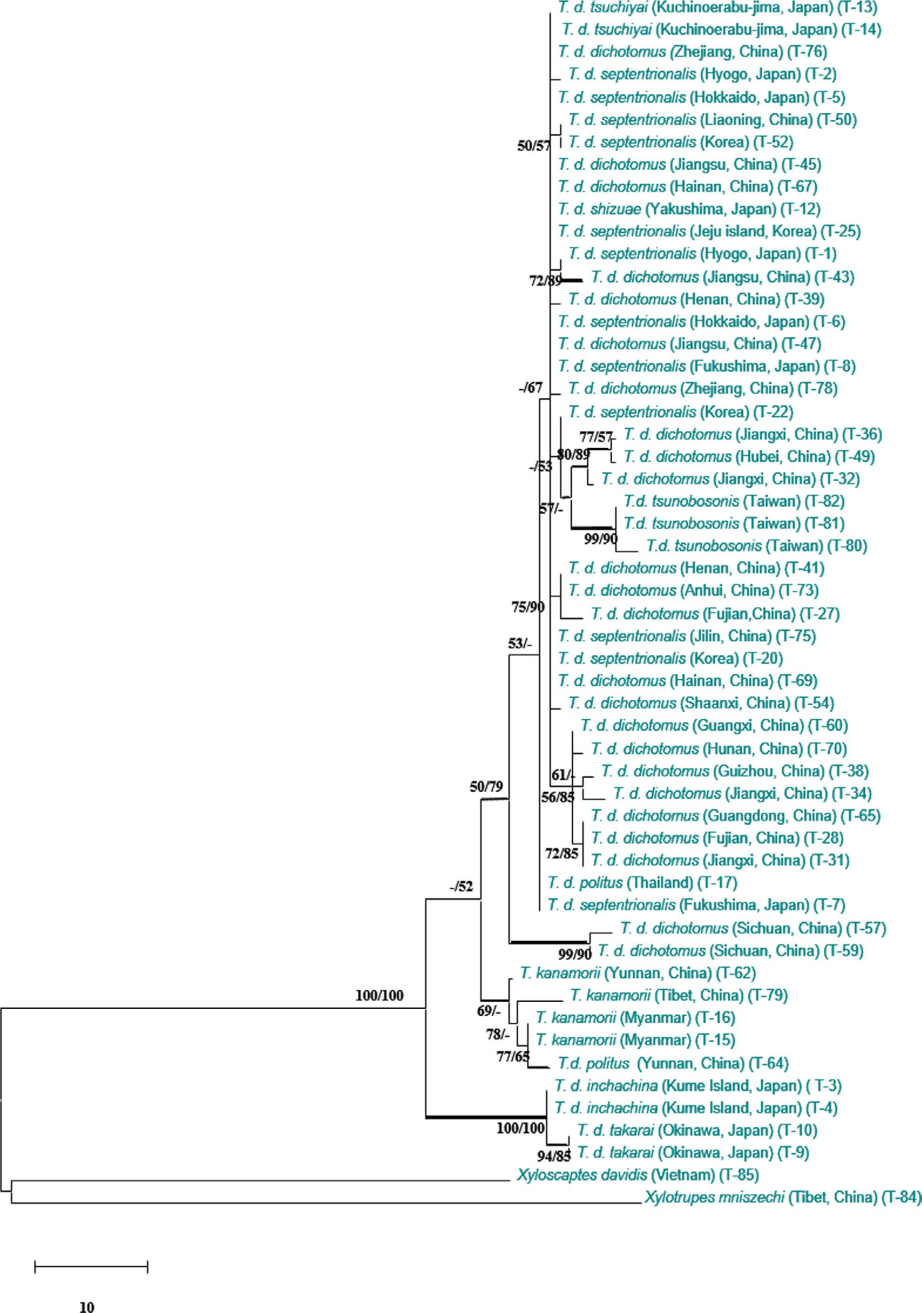
Phylogram of *Trypoxylus* species based on 16S rRNA and COII gene. MP and ML bootstrap support values above 50% are shown at the first and second position. Thickened branches represent posterior probabilities above 0.95 from BI

To assess the monophyly of the *Trypoxylus* subspecies, a phylogenetic analysis was performed based on combined 16S and COII sequence dataset (Figure 2). The alignment included 51 *Trypoxylus* ingroup sequences that when aligned were 1,239 characters in length including gaps, with 1,042 characters monomorphic, another 113 variable characters were parsimony‐uninformative, and 84 characters were variable and parsimony‐informative. MP analyses generated 12 most parsimonious trees of equal length with the following parameters: tree length (TL) = 274; consistency index (CI) = 0.818; retention index (RI) = 0.839; and rescaled consistency index (RC) = 0.686. ML and BI trees were similar in topology to the MP tree.

The phylogenetic tree based on 16S and COII sequence dataset did not clearly resolve the relationships among the *Trypoxylus* subspecies (Figure 2), as representatives of *T. d. dichotomus* and *T. d. septentrionalis* from Korea and different parts of China formed a clade with weak branch support. However, the 16S and COII data supported the sister relationship between *T. d. takarai* (Okinawa T‐9, T‐10) and *T. d. inchachina* (Kumejima T‐3, T‐4; branch support for the two subspecies MP/ML/BI = 100/100/1) in Japan, as well as a clade of *T. kanamorii* and *T. d. politus* from Myanmar and China.

### 3.3 SLAF sequencing and SNP discovery

Using the genome of mountain pine beetles *D. ponderosae* (Coleoptera: Scolytidae) as a reference, the restriction fragment length of RsaI‐HaeIII, ranging from 264 to 364 bp, was used for defining SLAF tags in our study taxa. Polymorphism analysis of the SLAF markers was summarized in Table 4. Fifty‐three DNA libraries were sequenced using the SLAF‐seq technique, which generated 114.16 Mb of data, ranging from 816,730 to 9,167,064 reads for each library. In all libraries, 92.20% of bases had high‐quality Q30 values. The Q30 data of tested sequences were higher, indicating that the base error rate was very low, and thereby the sequencing results were reliable. Major characteristics of the 53 SLAF‐seq libraries were summarized in Table 4. The average guanine–cytosine content was 35.28%, with each library ranging from 36.24% to 53.77%. The average depth of sequencing obtained was 17.63, ranging from 6.09 (in T‐67) to 109.26 (in T‐25) for each DNA library. Additionally, the total number of SLAF tags was 1,374,985. The highest number of SLAFs by individual was 245,614 in library T‐80, whereas the lowest one was 13,864 in library T‐22, whose depth was 59.12. Out of all tags identified, 330,799 were polymorphic SLAF markers.

**Table 4:** Polymorphism analysis results of the SLAF markers of *Trypoxylus*

Feature	Value
No. of reads	114.16 Mb
Average Q30 percentage	92.20%
Average GC percentage	37.42%
Average depth in individuals	17.63
Total number of SLAFs	1,374,985
No. of polymorphic SLAFs	330,799
No. of SNPs	2,127,917
No. of SNPs (MAF > 0.5)	46,939

SNP markers were developed based on high depth mapped to the reference genome in each SLAF tag. We identified 2,127,917 SNPs, with an integrity score between 30.16% and 53.93%. To avoid bias in the estimation of the baseline differentiation and to reduce sequencing and PCR error from the SNPs dataset, SNPs with a minor allele frequency (MAF > 0.05) on average across sampling locations were retained and used for analyses. It has been shown that very low‐frequency SNPs (MAF < 0.05) created biases in quantifying genetic connectivity and should therefore be excluded when inferring demographic processes (Roesti et al., 2012). With these criteria, a total of 46,939 high‐integrity SNPs with an MAF > 0.05 were identified among 53 individuals. There was a broad difference in SNP heterozygosity among different samples, with the number of heterozygous SNPs before filtering ranging from 21,157 in T‐20 to 1,188,339 in T‐80. The filtered SNP dataset provided sufficient information to detect the genetic structure and genetic diversity of *Trypoxylus* taxa.

### 3.4 Phylogenetic analysis and genetic structure based on SLAF‐seq derived SNPs

**Figure 3:**
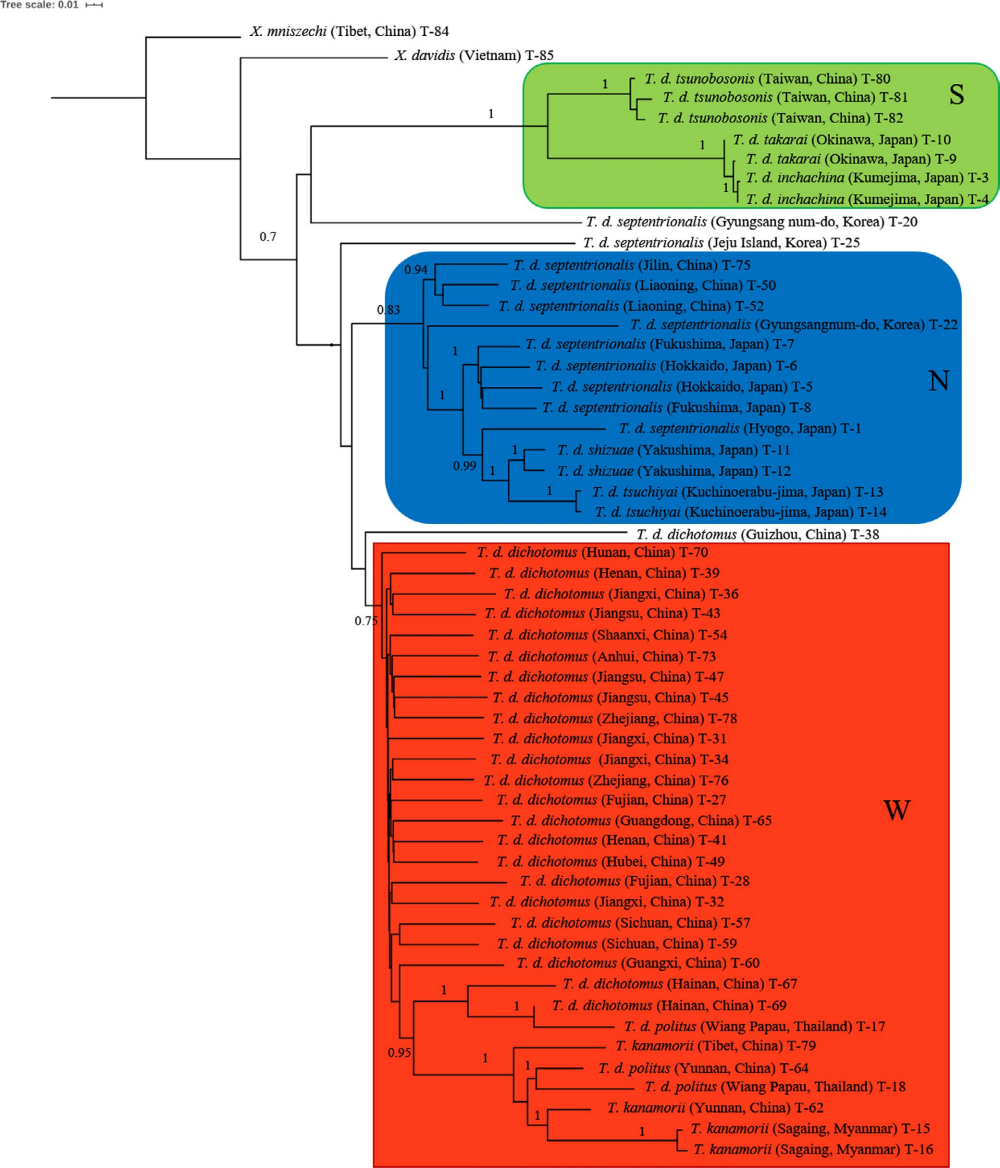
Phylogenetic tree of *Trypoxylus* subspecies based on the analysis of 46,939 SNPs developed from SLAF. Three major groups were colored

Phylogenetic analyses were conducted to determine the relationships among 53 specimens of *Trypoxylus* subspecies in different regions of Asia (Figure 3). Three well‐supported genetic clusters (North, South, West) were recognized. Genetic clusters North contained individuals from Northeast China (Jilin, Liaoning), mainland of Japan (Fukushima, Kuriyama, Hyogo, Yakushima, Kuchinoerabu‐jima), Korean Peninsula and Jeju Island of Korea; West was made up of individuals from Central and Southern China, including Tibet, Hainan Island, Thailand, and Myanmar, with the remaining individuals from Taiwan (China), the islands of Okinawa and Kumejima (Japan) placed in the South genetic cluster.

There were discrepancies between the phylogenetic groups and subspecies established with traditional morphological studies; some subspecies were monophyletic, but some were not. Two subspecies in South were monophyletic; *T. d. inchachina* from Kumejima, Japan, and *T. d. takarai* from Okinawa, Japan, were closely related to each other, and together, they formed a sister relationship to *T. d. tsunobosonis* sampled from different locations in Taiwan (Figure 3).

Within group North, *T. d. shizuae* from Yakushima and *T. d. tsuchiyai* from Kuchinoerabu‐jima in Japan were in a sister relationship; however, they were more closely related to *T. d. septentrionalis* population in Japan than other *T. d. septentrionalis* population from Korea and Northeastern China. Three samples of *T. d. septentrionalis* from Northeastern China were closely related to each other. In group West, *T. kanamorii* from Myanmar, Tibet (China) and Yunnan (China) were clustered together with *T. d. politus* and *T. d. dichotomus* from Thailand, Yunnan (China), Hainan (China). These samples also clustered with *T. d. dichotomus* from Sichuan and Guangxi in China. The rest of samples from Eastern China formed a paraphyletic clade. Two *T. d. septentrionalis* (T‐20, T‐25) from Korea, and one *T. d. dichotomus* (T‐38) did not cluster with these three genetic clusters and may represent hybrid or relictual lineages.

**Figure 4:**
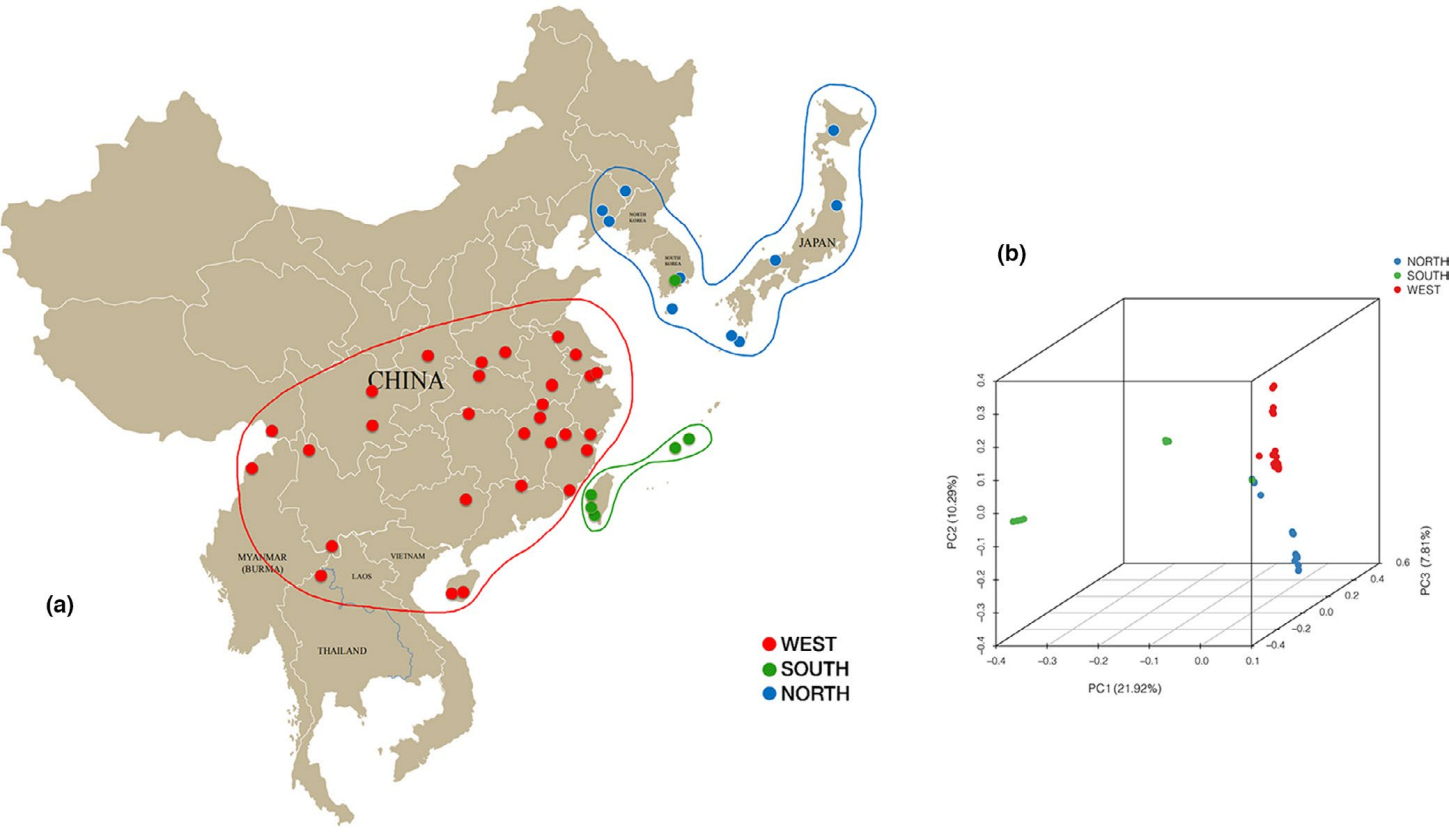
Genetic structure of *Trypoxylus* species in East Asia. (a) Sampling locations and the Admixture result of three populations. (refer to Table 2 for population abbreviations). (b) PCA analysis of *Trypoxylus* species depicting the genetic structure

PCA also separated specimens into three major clusters (Figure 4a,b), which was similar to the phylogenetic analysis based on identified SNPs. PCA spatially placed the group South on the left bottom quadrant of the first PCA axis (account for 22.14% of variation), while the other two groups clustered within the right quadrant of the first PCA axis (Figure 4b). Considerable divergence was also illustrated within group South, in which samples from Taiwan and southern Japan (Okinawa and Kumejima) could be further divided into two well‐supported clades. The North population was slightly differentiated from the West population along the third PCA axis (7.89% of the variation; however, they were apparently separated along the second PCA axis (10.4% of the variation).

**Figure 5:**
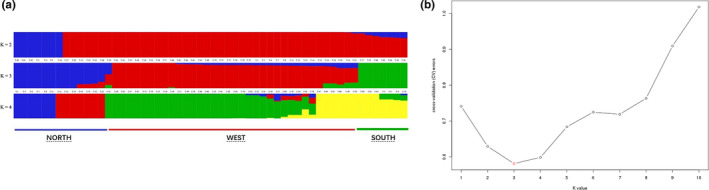
Genetic structure of *Trypoxylus* inferred by ADMIXTURE (*K = *2 to 4). Population codes are given in Table 2

ADMIXTURE analysis generated similar genetic clustering patterns to those based on phylogenetic analysis of SNPs and PCA. The estimated membership fractions of 53 specimens for different values of *K* were assessed across a range from 1 to 10, with the optimum cluster value of 3 (*K* = 3) determined using the cross‐validation error rate (Figure 5), which indicated that the sampled specimens could be categorized into three genetic groups. Group North contained 14 specimens, which were from Northeastern China (Jilin, Liaoning), mainland of Japan (Fukushima, Kuriyama, Hyogo, Yakushima, Kuchinoerabu‐jima), Jeju Island (South Korea) and the Korean Peninsula. Group West contained 32 specimens, most of which were from different provinces in China, including central China, Southwestern China, Southeastern China, Tibet, and Hainan Island, while the remainder were from Thailand and Myanmar. Group South contained 7 specimens, 3 of which were from Taiwan, and the rest were from Okinawa, and Kumejima of Japan. Two specimens (T‐25, T‐20) appeared to have admixed origin.

### 3.5 Genetic diversity among populations

The genetic diversity of the three genetic clusters inferred by genome‐wide SNP data is presented in Table 5. Expected heterozygosity in the South genetic cluster was the highest (*H*_e_ = 0.338), followed by genetic cluster North (*H*_e_ = 0.356) and lowest in the West genetic cluster (*H*_e_ = 0.29). However, observed heterozygosity (South = 0.097, North = 0.197, and West = 0.177) was lower than expected in all genetic clusters suggesting past genetic bottlenecks or some other demographic or selective process is common in *Trypoxylus* beetles. The mean PIC also revealed that group South was highly polymorphic (0.281), whereas group West exhibited the lowest PIC (0.240).

**Table 5:** Genetic diversity metrics from genetic clusters determined in ADMIXTURE

Population	Observed allele number	Expected Allele number	Observed heterozygous number (*H*_o_)	Expected heterozygous number (*H*_e_)	Nei's diversity index	Shannon Wiener index	Polymorphism information content (PIC)
North	2	1.567	0.197	0.338	0.352	0.511	0.273
South	2	1.638	0.097	0.356	0.384	0.528	0.281
West	2	1.457	0.177	0.290	0.295	0.455	0.240
Total	2	1.392	0.111	0.257	0.260	0.414	0.216

### 3.6 Population divergence and gene flow

Analysis of molecular variance (AMOVA) indicated that 35.89% of the total variation attributed to variation among populations, while 28.29% and 35.82% were attributed to among individuals within populations and within individual, respectively (Table 6).

**Table 6:** Analysis of molecular variance (AMOVA) for *T. dichotomus* based on three major populations inferred from SNPs data and ADMIXTURE analysis

Source of variation	*df*	SS	Variance	%	*p*‐values
Among populations	2	145,731.77	2,607.16	35.89	<.001
Among individuals within populations	50	292,407.42	2,054.62	28.29	<.001
Within individuals	53	121,563.00	2,602.30	35.82	<.001
Total	105	559,702.19	7,264.07		

Note

Degree of freedom (*df*), sum of squares (SS), variance components (Variance), percentage of total variation (%) contributed by populations.

Pairwise *F*_ST_ revealed differing levels of divergence between North, West, and South genetic clusters. The highest differentiation was between genetic clusters North and South (*F*_ST_ = 0.345), while the least differentiation was between West and North (*F*_ST_ = 0.094) (Table 7), which was corroborated with the results based on SNPs.

**Table 7:** Pairwise *F*_ST_ among the three populations inferred from ADMIXTURE analysis

	South	West	North
South		0.174	0.345
West			0.094

**Figure 6:**
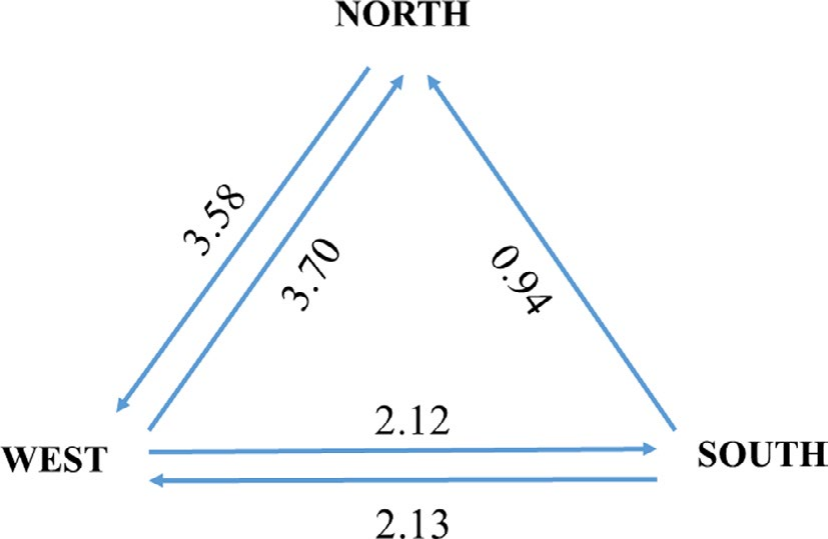
Migration rate estimates among three population groups. The arrow represented the direction of geneflow

In addition, the gene flow and migration rates among populations were estimated using MIGRATE‐N. Based on the Bezier approximation scores in model selection, the following gene flow rates and directionality were inferred (Table 8). Bidirectional gene flow between North and West as well as between South and West genetic clusters was inferred. The rate of bidirectional gene flow was similar between these genetic clusters, but the rates were different when compared to different genetic clusters as the average rate of gene flow (*N*_m_) between North and West genetic clusters was 3.70 and 2.13 between South and West genetic clusters. Gene flow was inferred to be unidirectional from the South to North genetic cluster at a rate of *N*_m_ 0.94 (<1), and the genetic differentiation (*F*_ST_ value = 0.345) between these genetic clusters was also inferred to be the highest among the clusters we compared. As such, a strong barrier to gene flow was inferred between the two population groups (Figure 6).

**Table 8:** The estimates of migration rate among three populations using Migrate‐N

Group	Model	Bezier approximation score (1b)
NORTH‐SOUTH	Mo1	−60,030.15
	Mo2	−59,873.85
	Mo3	−61,398.83
	Mo4	−60,560.66
NORTHWEST	Mo1	−138,945.00
	Mo2	−141,897.74
	Mo3	−139,602.63
	Mo4	−141,312.02
SOUTHWEST	Mo1	−120,988.34
	Mo2	−124,061.54
	Mo3	−122,687.21
	Mo4	−123,467.90

## 4 DISCUSSION

### 4.1 Population structure and biogeography of *Trypoxylus*

Resolving patterns of intraspecific variation such as genetic diversity, populations genetic structure, and gene flow within and among populations are essential to understand historic processes such as migration, isolation, and adaptive radiation (Mendelson & Shaw, 2005). Furthermore, patterns of genetic partitioning and divergence when compared to abiotic and biotic landscape features can be an effective method in finding correlations and from there testing hypotheses regarding processes that may have shaped the observed patterns. We investigated the population structure of *T. dichotomus* and related species using high density SNP markers covering the whole genome, as well as sequences from 16S rRNA and COII. Phylogenetic analysis using the SNP dataset, in addition to ADMIXTURE and PCA analyses, revealed the presence of three genetic groups in the *Trypoxylus* subspecies/populations.

The genetic partitioning of individuals is clearly related to geographic distribution. For instance, the South genetic cluster is made up of individuals found on the islands of Taiwan, Okinawa, and Kumijima. The phylogenetic branching pattern within this genetic cluster is consistent with the pattern expected for an island radiation wherein individuals from the same island cluster together and the distance of islands from one another is reflected in branching order (i.e., ancestors from islands nearby to each other coalesce before coalescing to ancestors from more distant islands) (MacArthur & Wilson, 1967; Wilson, 1959; Zimmerman, 1970). From the branching pattern, it appears that Taiwan was colonized first, and then Okinawa followed by Kumejima. The subspecific designations in this genetic cluster/clade follow the pattern of geographic/island distribution suggesting that morphological traits associated with subspecific designations may also be involved with adaptive traits which evolved after island colonization (e.g., Darwin's finches). The T‐20 individual, which is a member of the South genetic cluster, does not follow the island model as it was close to the individuals in the North cluster on the Korean peninsula. Possible explanation for the presence of T‐20 on the Korean peninsula could be a recent dispersal from one of the islands or T‐20 may represent an ancient or admixed genotype. The North and West genetic clusters are made up of individuals that are geographically adjacent and from a similar set of subspecific taxa. Branching patterns within these clusters further resolve the pattern of genetic partitioning correlating with geography. For instance, individuals (*T. kanamorii*, *T. d. dichotomus*, and *T. d. politus*) from the southern part of West genetic cluster form a well‐supported clade within the West genetic cluster suggesting a recent radiation with subsequent isolation. In general, the partitioning of individuals into North and South genetic clusters follows previous observations for *Trypoxylus* populations).

Historic and ongoing gene flow reduces differentiation between populations (Ellstrand & Elam, 1993). The high differentiation and relatively low level of migration from genetic cluster South to North and West indicate barriers to gene flow. The geographic distance from Taiwan to mainland China (closest geographic distance between the South and West genetic clusters) is only about 130 km, while the distance from Taiwan to Okinawa is about 700 km. Given that individuals on Taiwan are more closely related to individuals on Okinawa than they are to individuals on mainland China, suggests that dispersal from the mainland to islands is rare and may be more strongly influenced by ocean currents (Karl, 1999) than geographic proximity. While island dispersal has occurred in both North (Japan and associated islands and Jeju, Korea) and West (Hainan island, China) genetic clusters, these dispersal events are inferred to be more recent and/or include ongoing migration as individuals on these islands did not resolved in separate genetic clusters. However, branching patterns in the phylogenetic analysis (Figure 3) suggests subclustering of island lineages within North and West genetic clusters. Additionally, gene flow between North and West genetic clusters is more frequent, possibly because of fewer geographic barriers.

In all genetic clusters, the observed heterozygosity was lower than expected heterozygosity suggesting that demographic and/or behavioral processes might be decreasing genetic diversity in *Trypoxylus* populations. It is well known that male *Trypoxylus* beetles compete against one another for access to females (Hongo, 2003, 2007). These types of competitive behaviors have been associated with increases in heterozygosity in other animal species (e.g., Bensch et al., 2006; Seddon et al., 2004). However, founder effects associated with colonizing new territory have been shown to strongly decrease observed heterozygosity in animal populations (e.g., Kekkonen et al., 2012; Keller & Waller, 2002). The greatest difference between observed and expected heterozygosity was found in the South genetic cluster which is consistent with the inferred founder effects associated with island dispersal. More work is needed to elucidate what factors are contributing to the low observed heterozygosity found in this study.

Despite the geographic isolation of various subspecies/population, some individuals were inferred to be of admixed origins (Figures 3 and 4). Given the popularity of keeping *Trypoxylus* beetles as pets and importance in medicine, they have been translocated throughout Asia. Beetles kept as pets or used in breeding are often released back into the wild (Nagai, 2007). Through such actions, beetles from divergent lineages may be brought into contact and reproduce. Translocation and release into the wild may explain the presence of a South genetic cluster genotype (T‐20) found in the geographic range of North genetic cluster individuals. Translocation and release into the wild can have detrimental effects on wild locally adapted populations through outbreeding depression (Frankham, 2010) and should be studied further especially among isolated island populations. *T. d. septentrionalis* samples from Korea were quite divergent as isolates from Jeju Island (T‐25) and Gyungsang num‐do (T‐20) did not cluster with beetle populations from Northeastern China and Japan. They could represent different independent lineages as a result of historic separation or recent geographic isolation.

### 4.2 Taxonomic implications

Ten recognized subspecies of *T. dichotomus* and closely allied species *T. kanamorii* have been described from different parts of Asia, including the countries China, Japan, Korea, Myanmar, Thailand, Laos, and India (Adachi, 2017; Kôno, 1931; Kusui, 1976; Li et al., 2015; Linné, 1771; Nagai, 2006, 2007; Prell, 1934; Satoru, 2014). Taxonomic boundaries among these subspecies were not clearly defined in the past. We attempted to resolve the phylogenetic relationships among *Trypoxylus* subspecies based on 16S and COII sequences, which have provided phylogenetic resolution for delineating Coleoptera genera in past studies (Lee et al., 2015). However, 16S and COII did not resolve *Trypoxylus* subspecies delineations with high support.

Using broadly distributed SNP markers, subspecies were resolved in three well‐supported genetic clusters that generally corresponded to geographic origins. Although *T. dichotomus* and related species share similar morphological features (body size, lustrousness, cuticle color, and distribution of pubescence; Table 2), none of the analyzed morphological characters taken individually and commonly used in the traditional taxonomy of *T. dichotomus* proved to be unambiguously species‐ or subspecies‐specific. The overlap of some characters in *Trypoxylus* may be the result of phenotypic plasticity, not previous observed in taxonomic descriptions, resulting from the interactions between genetic, environmental conditions (e.g., temperature or latitude), and/or biotic factors seen in other species (Davis et al., 2008; Gross et al., 2004). However, according to Buchalski et al. (2019), *T. dichotomus* populations in Kyoto and Hokkaido (in central and Northern Japan, corresponding to North group in this study) had significantly longer horns for a given body size, than those specimens in Taiwan and Yakushima (largely corresponding to group South) in this study.

Several subspecies circumscribed by morphological features were supported by the molecular data in this study. The best example was *T. d. tsuchiyai* and *T. d. shizuae* from Japan that were resolved as monophyletic with high branch support (PP = 1). Both subspecies were similar to *T. d. septentrionalis*. However, *T. d. tsuchiyai* was smaller and wider in both sexes, with pronotum and middle frontal areas of elytra clothed with pubescence, and the female individuals had shorter and coarser pubescence on pygidium comparing to *T. d. septentrionalis*. *T. d. shizuae* lacked an obvious presence absence morphological trait but was different in body size ratio to *T. d. septentrionalis* among individuals examined for this study. Based on our results, *T. d. tsuchiyai* and *T. d. shizuae* should be retained as subspecies and considered for elevation to species following further study including genitalic dissections. *T. d. septentrionalis* is paraphyletic with the inclusion of *T. d. tsuchiyai* and *T. d. shizuae*. However, the resolution of several well‐supported clades within the North genetic cluster suggests that *T. d. septentrionalis* should be reexamined and possible refined to account for these well‐supported clades.

Both *T. d. inchachina* and *T. d. takarai* have been reported from the Okinawan Islands (Japan), and together, they formed a monophyletic clade with small genetic distance. *T. d. takarai* has been separated from *T. d. inchachina* as being a smaller and black bodied beetle, with shorter pronotal horns on male individuals. However, these two subspecies were found to be very similar to each other in morphological traits (Nagai, 2007). Compared with *T. d. septentrionalis*, they were both smaller in body size and possessed smaller cephalic/pronotal horns and were darker in body color in male individuals. Differences between these two subspecies were indistinguishable in smaller individuals. *T. d. inchachina* and *T. d. takarai* may be considered as independent lineages possibly because of allopatry. From Taiwan, *T. d. tsunobosonis* resolved as monophyletic and should be retained as subspecies and studied further for possible elevation to species. *T. d. tsunobosonis* had thinner pronotal horn in male individuals than *T. d. septentrionalis,* which was similar to *T. d. takarai* in male genitalia (Nagai, 2007).

The West genetic cluster comprised of 23 specimens of *T. d. dichotomus*, which was resolved as paraphyletic basal to *T. kanamorii* and *T. d. politus. T. d. politus* nested within. *T. d. dichotomus* is separated from *T. d. septentrionalis* by having a longer, thinner, and less glossy body as well as a longer thicker cephalic horn. However, specimens from Southern China and Central China did not show any significant difference in morphology. Within the paraphyletic grade of *T. d. dichotomus,* a well‐supported clade containing the subspecies *T. d. politus*. and the species *T. kanamorii* was resolved. Morphologically, *T. kanamorii* is very similar to *T. d. politus,* but it can be distinguished from the later in having a slender and black body, parallel‐sided elytra, shorter horns on head and pronotum, and slight pubescence on elytra (Nagai, 2006). The clade containing *T. kanamorii* is paraphyletic with respect to *T. d. politus*. As such the designation of “species” does not appear to be valid for *T. kanamorii*. Furthermore, if strictly applying a phylogenetic approach to taxonomic designations (de Queiroz & Gauthier, 1990), the recognition of clades (like that containing *T. kanamorii*) that result in the creation of basal paraphyletic grades does not adhere to the goal of species delineations recognizing monophyletic groupings.

## 5 CONCLUSION

Our study demonstrated that SLAF‐seq derived markers outperformed 16S and COII sequences in beetle taxonomy and provided improved resolution of the genetic differentiation of rhinoceros beetle populations from a large part of the species’ range. Phylogenetic analysis of SNPs indicated the presence of three distinct genetic groups, suggesting that the various subspecies fall into three distinct and well‐supported lineages. PCA and ADMIXTURE analysis also identified three genetic clusters (North, South, West), which corresponded to their origin, suggesting that geographic factors were important in maintaining within population homogeneity and between population divergence. Further study including more detailed morphological work, increased taxon sampling, and taxonomic circumscription is needed to improve the current systematics of *Trypoxylus* beetles. The current study provides an important dataset, analyses, and method for genotyping (SLAF‐seq) that can be used and built upon to answer unresolved questions of systematics, character evolution, chemical and immunological chemistry, and phylogeography in *Trypoxylus* and related taxa.

## CONFLICT OF INTEREST

All authors declare that they have no competing interests.

## AUTHOR CONTRIBUTION

**Huan Yang:** Data curation (equal); Formal analysis (equal); Writing‐original draft (equal). **Chongjuan You:** Data curation (equal); Funding acquisition (lead); Methodology (equal); Writing‐original draft (equal). **Clement Tsui:** Investigation (equal); Methodology (equal); Writing‐review & editing (equal). **Luke Tembrock:** Writing‐review & editing (equal). **Zhiqiang Wu:** Writing‐review & editing (equal). **Depo Yang:** Supervision (equal).

## Data Availability

Data have been deposited at the Dryad Digital Repository: https://doi.org/10.5061/dryad.qv9s4mwcz.
